# Lymphedema

**Published:** 2013-01-29

**Authors:** Howard D. Wang, Sachin M. Shridharani, Anthony P. Tufaro

**Affiliations:** Department of Plastic and Reconstructive Surgery, The Johns Hopkins University School of Medicine, Baltimore, Md

**Figure:**
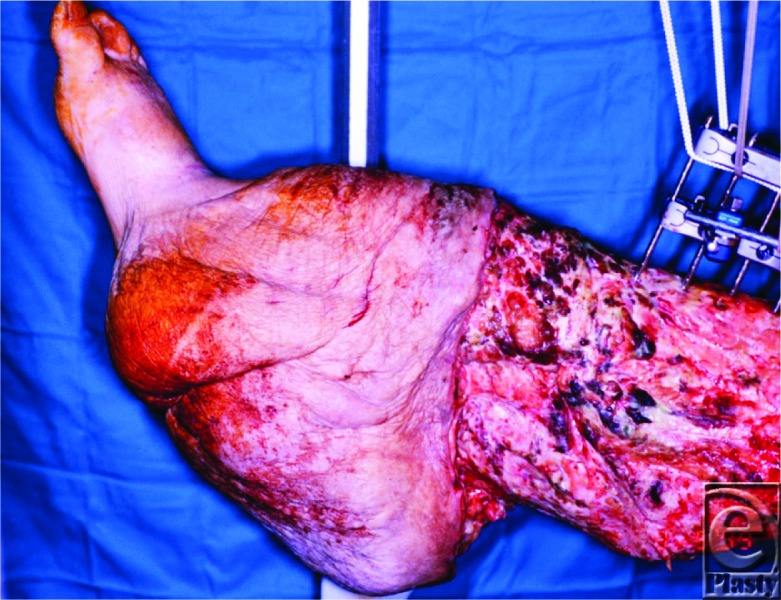


## DESCRIPTION

A 58-year-old man with a history of surgery for traumatic injury to his right lower extremity developed extensive swelling of the affected extremity consistent with lymphedema after the operation. He presented to the plastic surgeon with continued worsening of the swollen extremity after failing conservative management.

## QUESTIONS

1. **What is the most likely etiology of lymphedema in this patient?**
2. **What is the most common etiology of lymphedema worldwide and in the United States?**
3. **Describe the normal drainage anatomy of the lymphatic system.**
4. **How is lymphedema diagnosed?**
5. **What are the non-surgical therapies used to treat lymphedema?**
6. **What surgical approaches are available for the treatment of lymphedema refractory to conservative therapy?**

## DISCUSSION

This patient developed severe swelling in his right lower extremity shortly after his initial injury and multiple operative debridements of the affected extremity. The etiology of this patient's lymphedema is most likely traumatic disruption of the lymphatic channels. Common sources of lymphedema can be divided into primary and secondary causes. Primary lymphedema, often congenital, occurs because of malformation of the lymphatic system during late stages of lymphangiogenesis. Secondary lymphedema is caused by disruption of the lymphatic system and can result from malignancy, surgery, infection, trauma, or radiation. Worldwide, the most common etiology of lymphedema is “filariasis.” This frequently is a result of infection by the nematode *Wuchereria bancrofti*. In contrast, the vast majority of cases in the United States are related to malignancy and associated treatments.^1^

Lymphedema is a chronic and progressive disease marked by accumulation of fluid in the interstitial space. Chronic accumulation of interstitial fluid elicits an inflammatory response that leads to fat deposition and tissue fibrosis.^1^ Sequelae related to lymphedema include severe swelling, fibrosis, infections, and functional deficits. Chronic lymphedema can lead to elephantiasis, which is marked by massive enlargement of the affected region, brawny and thickened skin that resists wrinkling, and presence of verrucous papules and nodules.^2^ All these complications occur because the lymphatic system's ability to perform its important functions is impaired.

The lymphatic system is found throughout the body and serves both immunologic and circulatory functions. Present in the skin and nearly every internal organ, lymphatic capillaries are thin, relatively large vessels composed of a single-layer of endothelial cells. Their main function is to return excess interstitial fluid and proteins to circulating blood. Lymph travels from lymphatic capillaries to larger collecting lymphatic vessels. These vessels have a smooth muscle cell layer to propel forward flow of the lymph; in addition, valves are present to prevent backflow.^3^ The collecting lymphatic vessels eventually drain into the thoracic duct, which originates at the cisterna chyli, located in the abdomen right of the midline. The thoracic duct travels vertically and terminates at the confluence of the left internal jugular and subclavian vein, where lymph enters the circulating blood.^4^ Disruption of this intricate system leads to lymphedema.

The diagnosis of lymphedema is typically made with history and physical examination alone. Patients present with swelling of the affected region. Initially, the edema is pitting, then nonpitting, and finally increasingly indurated. Skin changes such as peau d'orange, dermatitis, hyperkeratosis, and ulceration may be present. Stemmer sign, inability to lift the skin on the dorsum of the second digit of the foot, may also be noted. If the diagnosis is uncertain based on clinical findings, imaging studies may be obtained for confirmation. The criterion standard for evaluation of lymphedema is lymphoscintigraphy, a relatively noninvasive procedure in which 99mTc-labeled protein is injected intradermally at the distal aspect of the edematous region to visualize the lymphatic vasculature.^5^ Further imaging with duplex ultrasonography, computed tomography, or magnetic resonance imaging may be indicated to provide additional anatomic information or to evaluate for other disease processes such as deep venous thrombosis or malignancy.

Once the diagnosis is made, the goals of therapy include the following: decrease the edematous region, reduce risk of infection, and improve aesthetic appearance and function. Lymphedema is initially managed with conservative therapy, the mainstay of which is complete decongestive therapy (CDT), or also known as decongestive lymphatic therapy. Compression is at the core of CDT; however, this multimodality therapy also includes manual lymph drainage, physical exercises, skin care, multilayer compression bandaging, and elastic compression. Patient education regarding self-management is vital to the success of CDT as a lifelong maintenance phase is required after the initial treatment phase.^6^ Prior to initiating CDT, the patient should understand that this intensive therapy may alleviate their symptoms but does not cure the underlying lymphatic dysfunction. The effectiveness of CDT is variable and thought to depend on the degree of fibrosis present. In addition to physical therapy, pharmacologic agents may be employed. One commonly used drug is benzopyrones, which acts by stimulating proteolysis to reduce the volume of high protein edema.^7^ In cases of lymphedema that are refractory to conservative therapy, surgical consultation is the next step of management.

Several surgical options are available, including excisional procedures, bypass operations, and tissue transfers. Excisional procedures involve removal of the excess skin and subcutaneous adipose and fibrous connective tissue through surgical resection. Removal of subcutaneous edematous tissue may also be achieved with liposuction. Bypass operations are performed to reconstruct the disrupted lymphatic system via microsurgical anastomoses between lymphatic channels and veins, lymph nodes and veins, and distal and proximal lymphatic vessels. Lymphatic grafting and transfer of muscle and skin flaps have also been proposed as surgical options for the treatment of lymphedema. Comparing the reported outcomes of these 3 categories of surgical procedures, the greatest reduction in volume and circumference occurs after excisional procedures.^6^ Regardless of the choice of operation, surgical therapy should be combined with postoperative use of compression garments and CDT for long-term maintenance.

**Figure 1:**
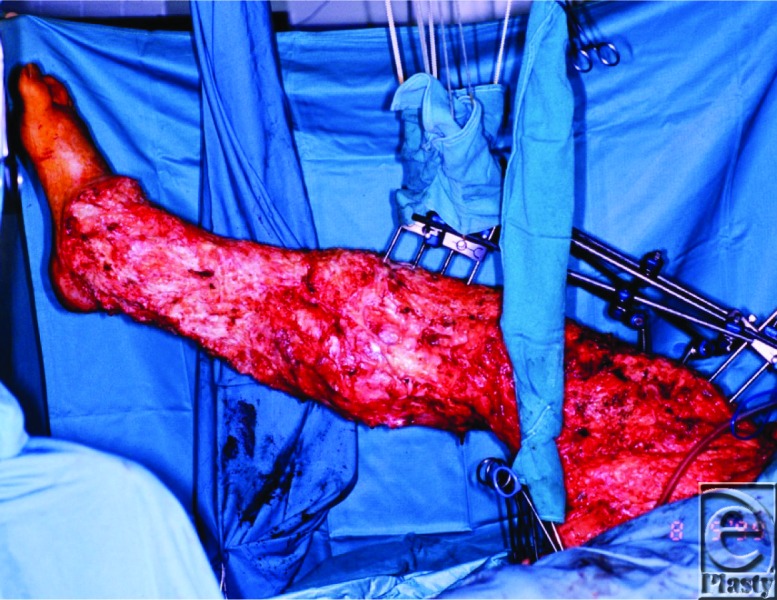
Intraoperative image of the affected limb after excision of skin and edematous tissue.

The patient in our case underwent the Charles procedure, named after sir Richard Henry Havelock Charles. The operation entails resection of all overlying skin and soft tissue superficial to the deep fascia in the region with lymphedema (Fig 1). The exposed area is then covered with skin graft collected from the removed tissue.
